# Assessment of Esophageal Shifts during Catheter Ablation of Atrial Fibrillation Using Intracardiac Ultrasound Integrated with 3-Dimensional Electroanatomical Mapping System

**DOI:** 10.3390/jcdd11040110

**Published:** 2024-03-31

**Authors:** Andrej Pernat, Mark Zavrtanik, Antonio Gianluca Robles, Silvio Romano, Luigi Sciarra, Bor Antolič

**Affiliations:** 1 Department of Cardiology, University Medical Centre Ljubljana, 1000 Ljubljana, Slovenia; mark.zavrtanik@kclj.si (M.Z.); bor.antolic@kclj.si (B.A.); 2 Department of Life, Health and Environmental Sciences, University of L’Aquila, 67100 L’Aquila, Italy; silvio.romano@univaq.it (S.R.); luigi.sciarra@univaq.it (L.S.); 3 Department of Cardiology, “L. Bonomo” Hospital, ASL BAT, 76123 Andria, Italy

**Keywords:** atrial fibrillation, catheter ablation, esophagus, atrioesophageal fistula, intracardiac echocardiography, three-dimensional electroanatomic mapping system

## Abstract

*Purpose:* Atrioesophageal fistula is one of the most feared complications of radiofrequency catheter ablation (RFCA) of atrial fibrillation (AF) as it is associated with high mortality. Determining the esophagus location during RFCA might reduce the risk of esophageal injury. The present study aims to evaluate the feasibility of using intracardiac echocardiography integrated into a 3-dimensional electroanatomical mapping system (ICE/3D EAM) for the assessment of esophageal position and shifts in response to ablation. *Methods:* We prospectively enrolled 20 patients that underwent RFCA of AF under conscious analgosedation. The virtual anatomy of the left atrium, the pulmonary vein (PV) ostia, and the esophagus was created with ICE/3D EAM. The esophageal positions were obtained at the beginning of the procedure and then after left and right PV isolation (PVI). Esophageal shifts were measured offline after the procedure using the tools available in the 3D EAM system. *Results:* Most esophagi moved away from the ablated PV ostia. After the left PVI, the median of the shifts was 2.8 mm (IQR 1.0–6.3). In 25% of patients, the esophagus shifted by >5.0 mm (max. 13.4 mm). After right PVI, the median of shifts was 2.0 mm (IQR 0.7–4.9). In 10% of patients, the esophageal shift was >5.0 mm (max. 7.8 mm). *Conclusions:* ICE/3D EAM enables the intraprocedural visualization of baseline esophageal position and its shifts after PVI. The shifts are variable, but they tend to be small and directed away from the ablation site. Repeated intraprocedural visualization of the esophagus may be needed to reduce the risk of esophageal injury.

## 1. Introduction

Radiofrequency catheter ablation (RFCA) is a well-established and widely adopted treatment option for patients with symptomatic atrial fibrillation (AF) [1]. RFCA is generally considered to be safe; however, the risk of complications is not insignificant. One of the most feared complications of the procedure is a thermal injury to the esophagus and the development of atrioesophageal fistula [2,3]. 

Due to its relative thinness and proximity to the esophagus, ablation on the posterior wall of the left atrium (LA) carries a risk of thermal injury to the esophagus [4]. Different approaches have been developed to visualize esophageal course during ablation to reduce the risk of injury. Three-dimensional electroanatomical image integration with preprocedural CT or MRI has been used [5,6]. However, the esophagus was shown to be a mobile structure, which may change position between preprocedural imaging and the time of the ablation procedure [7,8], making such an approach prone to errors. 

Only a few studies have evaluated intraprocedural imaging during RFCA for assessing esophageal course and revealed equivocal observations about its mobility [9,10,11]. In addition to fluoroscopy or esophageal probes to visualize the esophagus during the procedure, intracardiac echocardiography integrated with 3-dimensional electroanatomical mapping system (ICE/3D EAM) is a tool that enables virtual anatomy reconstruction of all relevant anatomical structures for AF ablation, including the esophagus [12]. 

The aim of our study was to evaluate the relationship between esophageal anatomical course and pulmonary veins and the extent of esophageal shifts in response to RFCA with intraprocedural imaging using ICE/3D EAM (CartoSound module, Biosense Webster, J&J, Diamond Bar, CA, USA).

## 2. Materials and Methods

### 2.1. Study Design and Patient Populations

We conducted a prospective study to evaluate the feasibility of ICE/3D EAM to assess esophageal shifts during RFCA for AF. Twenty patients (>18 years) with symptomatic AF that underwent RFCA were prospectively enrolled. All participants provided written informed consent. The study protocol complied with the Declaration of Helsinki and was approved by the Slovenian Medical Committee (Approval Number: 0120-64/2018/7).

### 2.2. Ablation Procedure and Mapping Protocol

**Figure 1:**
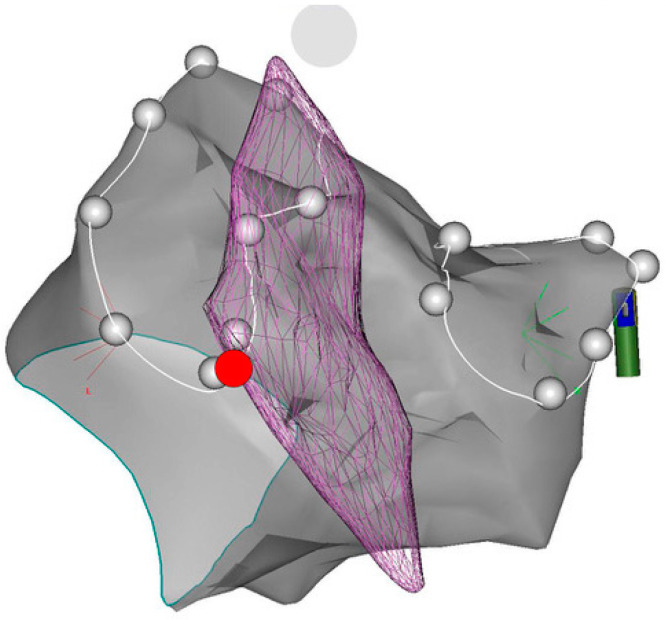
CARTO postero-anterior view of left atrium wall and esophagus. In this PA view, we can see a CartoSound-reconstructed virtual anatomy of LA (grey) and esophagus (violet), which overlies the left pulmonary vein ostia (white dots). SoundStar^®^ probe (green shaft, close to the RPVS) is visible on CARTO due to its magnetic sensor.

**Figure 2:**
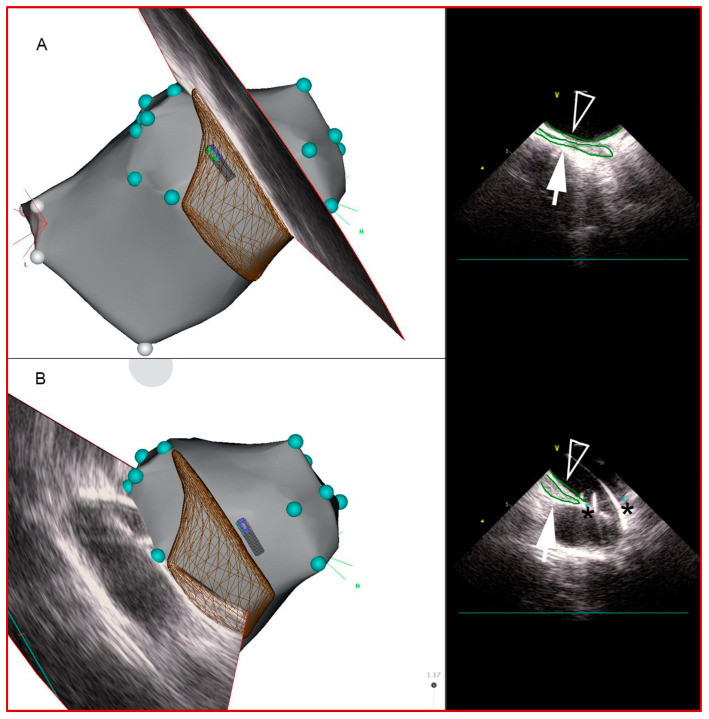
Sequential intracardiac echocardiography (ICE)-derived 2D images of esophagus on CARTO. Esophageal borders (green contours) were traced to create its 3-dimensional course over the LA posterior wall. Panels (**A**,**B**). Medial (rightward) and lateral (leftward) esophagus borders, respectively. In panel (**B**), ICE view, the guidewire is seen going into left inferior PV. Closed arrows: esophageal contours; open arrows: LA posterior wall contours; asterisks: PV ostia.

**Figure 3:**
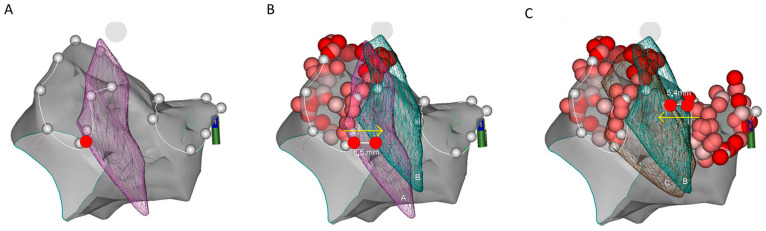
CARTO postero-anterior view showing virtual anatomy of LA (grey) and esophagus shift during ablation procedure. Panel (**A**). Esophagus (violet mesh) starting position: it overlies the left pulmonary vein ostia (white dots). Panel (**B**). Shifted esophagus (green mesh) after left PVI. Panel (**C**). Further esophagus shift (brown mesh) after right PVI.

Procedures were performed under conscious sedation using propofol and fentanyl. After venous accesses were obtained, intravenous heparin (100 IU/kg) was administered and continuously infused to obtain ACT > 300 s. An ICE probe (SOUNDSTAR^®^ Catheter, Biosense Webster Inc., Diamond Bar, CA, USA) was introduced into the heart and respiratory gating was performed. Afterward, the transseptal puncture was performed by ICE guidance and a long wire was placed into LA. The puncture site in the inter-atrial septum was dilated using long SL1 sheath that was then withdrawn to the right atrium (RA) with the wire left transseptally in LA. ICE probe was then introduced into LA along the wire. CartoSound virtual anatomy of the LA, the pulmonary vein ostia, the left atrium appendage, and the esophagus was reconstructed (Figure 1). The esophagus was imaged with ICE from the RA as well as from the LA, which allowed us to use multiple longitudinal as well as cross-sectional ultrasound planes of esophageal outer muscular layer for reconstruction of its virtual anatomy (Figure 2). After reconstructing virtual 3D anatomy, ICE probe was withdrawn to RA and long sheath reintroduced to LA over the wire. A decapolar circumferential catheter was inserted into LA, and, after second transseptal puncture, also an ablation catheter (Thermocool SmartTouch D or F, BiosenseWebster Inc., Diamond Bar, CA, USA). Contact force calibration was performed. RF delivery was performed point-by-point in power control mode according to CLOSE protocol aiming for a contiguous circle enclosing the ostia of the veins, regardless the esophagus position [13]. Specifically, in case of esophageal course just above the posterior lesion set line, Ablation Index was titrated up to 300 in case of pain. Esophageal course in relation to pulmonary vein ostia was constructed at three time points during the procedure: before any ablation was performed, after left, and after right PV ablation. We preferred ICE probe positioning in the LA due to superior visualization of pulmonary vein ostia and esophagus. Typically, after left PVI ICE probe was reintroduced into LA, a new course of the esophagus was constructed. The same was repeated following right PVI (Figure 3). 

After reconfirming PVI and before sheath withdrawal, possible pericardial effusion was evaluated by ICE. All procedures were performed without the use of fluoroscopy and without the use of esophageal temperature probe.

### 2.3. Esophageal Course Assessment and Data Analysis

All measurements were performed after the procedure on 3D map in postero-anterior projection in CARTO software (BiosenseWebster Inc., Diamond Bar, CA, USA). Prior to any ablations being performed, the position of the 3D reconstructed esophagus was determined. The initial esophageal location was classified as left-sided or right-sided whenever the esophagus passed within 5 mm from the respective PV ostium (PV ostia were defined and tagged with CartoSound). Otherwise, the position of the esophagus was defined as midline. For every reconstructed esophagus, we measured the distance between its ipsilateral border and the nearest corresponding PV ostium. Esophageal shifts were then assessed by measuring the differences in distance from esophageal position before and after ablations were performed with respect to the corresponding PV ostium. When ablating, the LPVs distance of the esophagus to the left PV ostium was measured, and conversely for the RPVs (Figure 3).

### 2.4. Statistical Analysis

We used descriptive statistics to present the baseline patient characteristics and results. Due to non-normal distribution of data, the range of the esophageal shifts is presented as median with interquartile range (IQR). Individual esophageal shifts are also illustrated graphically.

## 3. Results

The study population consisted of 20 patients; the median age was 61.0 years (IQR 51.8–66.0), and 75% were male. The clinical characteristics of each patient and AF type are summarized in Table 1. Of note, the most common indication for ablation was drug-resistant, symptomatic paroxysmal AF (75%); among them, a concomitant CTI dependent atrial flutter was present in 7 patients (35%). The remaining 25% of patients who underwent ablation had persistent AF.

The 3D reconstruction of esophagus was successfully obtained in all cases at all three time points (before ablation, after left PVI, after right PVI).

The initial esophageal position was most often left-sided in 55% of cases, followed by midline (30%), and right-sided (15%).

**Figure 4:**
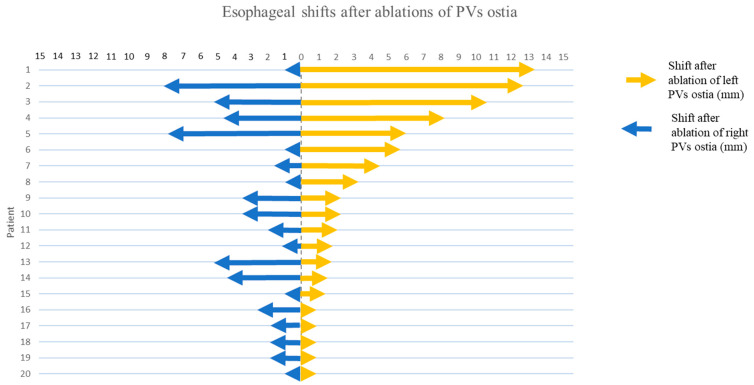
Extent of absolute esophageal shifts. On the Y-axis, each individual patient is represented (1–20). Arrows represent the differences in distance between the ipsilateral border of the esophagus and the corresponding pulmonary vein ostium after ablation.

In general, the esophagi moved to the side opposite the ablated PVs. Analyzing shifts after left PVI revealed that 60% of esophagi moved towards the right; the median shift was 2.8 mm (IQR 1.0–6.3) (Figure 4). In 5 patients, the esophagus moved more than 5 mm; in 3 patients, it moved more than 10 mm (maximal shift 13.4 mm). In contrast, we also noted a static position or only minimal shifts (<1 mm) of esophagi in five patients.

After right PVI, most esophagi moved towards the left (65%). The range of shifts was smaller than after ablation of LPVs with a median of 2 mm (IQR 0.7–4.9). In 2 patients the esophageal shift was more than 5 mm (maximal shift 7.8 mm). In 6 patients, we observed shifts less than 1 mm.

Successful PVI was achieved in all patients. No patient had major complications, including esophageal fistula, acutely or at follow up.

## 4. Discussion

The main finding of our study is that the esophagus is a mobile structure during RFCA for AF in consciously sedated patients. The shifts had unpredictable range, but in general they tended to be small and directed away from the ablation site. We provide a proof of utility for a non-invasive fluoroless methodology, such as ICE/3D EAM. This technology enabled intraprocedural virtual anatomy reconstruction of the esophageal course in all patients at multiple time points during the procedure, as well as precise evaluation of the relationship between the tip of the ablation catheter and esophageal wall.

Given the considerable risk of esophageal injury, especially the highly fatal atrioesophageal fistula, there is an unmet need for the development of the most appropriate strategy to avoid esophageal damage during RFCA.

In 2017 HRS/EHRA/ECAS/APHRS/SOLAECE consensus statement, different strategies on this topic were discussed and concluded that repeated imaging or visualization of esophagus seems to be a reasonable strategy [14]. Preprocedural imaging with CT/MRI is a common practice in many centers. However, it may not be sufficiently accurate in terms of obtaining esophageal location, mainly due to peristaltic movements that provoke changes in shape and location between the time imaging is performed and the time of the procedure. A study of 20 patients that compared esophageal position determined by CT (performed in the week prior to ablation procedure) with intraprocedural 3D/ICE imaging, found differences in position in 45% of cases [7]. Moreover, the esophagus may change the position also during the procedure as shown by our study. Earlier data on esophageal mobility during left atrial ablation procedures were somewhat contradictory. The first observation of the intraprocedural esophageal mobility during RFCA was provided by Good et al. in 2005 [9], and later confirmed by Daoud et al. [10]. However, Sherzer et al., in their study of 27 patients reported a static position of the esophagus during RFCA using an electrocatheter in the esophagus for continuous non-fluoroscopic localization [11]. This observation, opposite to our findings, is probably related to the use of general anesthesia, which may inhibit esophageal neural reflexes and consequently prevent contractions. Furthermore, even the presence of a stiff instrument in the esophagus such as an electrocatheter—or even a thermal probe—can limit both spontaneous and swallowing-related esophageal movements.

An important additional value of our study is the stepwise reassessment of esophageal position after ablating left and right PVs. We found that esophagus mostly responded in a predictive way—it moved away from the ablated PV ostia. Movements toward the ablation side were never seen. The observed esophageal response may convey an esophageal “defense mechanism” to avoid thermal injury, as an increase in esophageal temperature was shown to enhance the amplitude of peristaltic contractions, likely mediated by local neuromuscular reflexes [15,16]. A comparison of shifts after left-sided and right-sided ablation identified that esophagi shifted to a greater extent after ablation of LPVs. This observation may be due to a smaller distance between the esophagus and left PV ostia, as the initial position of most esophagi was left-sided. The distance between the esophagus and PVs is an important predictor of esophageal temperature rise [17]. Thus, this observation may further support the hypothesis of “temperature-induced” esophageal shifts. However, due to small sample size and the methodological limitations, such sub-analysis could not be obtained in the present study.

The observed esophageal shifts in our study were generally smaller than previously reported by Daoud and Good [9,10]. This difference could be related to different method of esophageal visualization. Both the above-mentioned studies used fluoroscopy with barium contrast to visualize esophagus course during RFCA. Residues of barium contrast that remained in esophagus after initial ingestion may elicit secondary peristaltic wave, even in the absence of deglutition, and therefore induce or magnify esophageal movements [18,19]. On the other hand, large heterogeneity in range of shifts among esophagi was common to all studies.

Another, even more important, limitation of barium esophagogram is its inability to obtain a detailed esophageal position. Fluoroscopy is not able to visualize the outer layer of esophagus, a part that is closest to the PV ostia, and thus the most relevant during ablation procedure. This limitation is also seen in fluoroless reconstructions of virtual esophageal anatomy using various electroanatomical catheters [11]. ICE/3D EAM used in our study overcomes these limitations, as its reconstructions rely on outlining the outer muscular layer. Hence, we believe that ICE/3D EAM arguably allows better assessment of esophageal position, and therefore provides additional anatomic detail that is crucial for the safe ablation.

Despite the considerable risk for esophageal injury, Kettinger et al. have shown, that exclusion of area adjacent to esophagus results in a higher rate of incomplete PVI and is associated with higher recurrence of arrhythmia after 3-year follow-up [20]. Accurate real-time visualization of esophagus therefore remains key to safe, but also for effective ablation, giving the possibility to tailor RF parameters when ablating close to esophagus. A recent randomized trial by Ye et al. provided evidence that ablation guided by soluble contrast for esophageal visualization reduces the incidence of ablation-related esophageal injury [21]. In our study ICE/3D emerged as a feasible tool for the real-time intraprocedural monitoring of esophageal position, which enables reconstruction of complex anatomical interrelation of esophagus and LA posterior wall.

Among the tools aiming to increase safety of RFCA regarding esophageal injuries, an active cooling tool (ensoETM, Attune Medical, Chicago, IL, USA) has been tested in single center and multicenter trials showing better safety and outcomes, and shorter duration procedure over a control group, with the limitations of requiring general anesthesia [22,23,24,25].

Recently, the EASY AF trial has been prematurely stopped because it showed a significant reduction of esophageal injuries—assessed with endoscopy—between a deviation device group vs. a control group [26]. This device has the advantage to effectively displace the esophagus away from the ablation site. However, it requires general anesthesia which is a significant draw-back for many EP laboratories.

Conversely, our approach does not require general anesthesia or deep sedation to enable placement of a thermal probe or an electrocatheter in the esophagus, as previously described [11], and it allows performing complete zero-fluoroscopy RFCA for AF. The lack of requirement for general anesthesia is not a trivial aspect since from the patient management point of view this reduces pre- and post-ablation preparation times, furthermore dedicated anesthesiologist is not necessary.

### Study Limitations

A small number of enrolled patients is the main limitation of our study. A larger patient population would enable us some sub-analysis that could further strengthen our observations. Although ICE/3D EAM appears to be a feasible tool that offers some advantages compared to other real-time visualization methods, our study was limited to observing instead of comparing different approaches of esophageal visualization. All patients enrolled in our study received conscious sedation during ablation and all procedures were performed without the use of fluoroscopy, according to current trends. The lack of a control group does not allow to compare procedural duration time with and without the use of ICE/3D EAM for esophagus reconstruction as well as the incidence of complications. However, we can state that introducing ICE probe in LA and reconstructing esophagus course is not time consuming (adds maximally a few minutes to the procedure duration), but time invested in using ICE could be pay off in greater safety.

In addition, the results of our study cannot be directly translated to other ablation approaches such as high power short duration ablation or other techniques including cryoablation PVI. Indeed, it was shown on animal models, that both energy source and ablation technique influence the risk for esophageal injuries [27].

Finally, the use of dedicated ICE probe which is compatible only with CARTO system may represent an important limitation for those EP labs having different 3D EAM systems.

## 5. Conclusions

Intracardiac echocardiography integration with 3-dimensional electroanatomical mapping system enables intraprocedural visualization of esophageal position and assessment of its shifts during radiofrequency energy delivery. In procedures performed under conscious analgo-sedation, esophagus shifts away from the ablation site, which may serve as a protective mechanism against excessive thermal injury.

## Figures and Tables

**Table 1:** Baseline patient characteristics.

Baseline Patient Characteristics	
Number of patients	20
Age (years)	61.0 (51.8–66.0)
Male	17 (85%)
Body mass index (kg/m^2^)	27.7 (24.8–31.7)
Atrial fibrillation type (%):	
Paroxysmal	75%
Persistent	25%
Concomitant CTI dependent atrial flutter (%)	35%
Antiarrhythmic drugs (%):	
Beta blockers	50%
Propafenone	15%
Sotalol	10%
Amiodarone	35%
LAVI *	45 mL/m^2^ * (43–50)
Procedural time (“skin to skin”)	223.68 min (187.5–255)

Values are presented as median with interquartile range and as percentage (%). * LAVI: left atrial volume index (available only for 5 patients).

## Data Availability

Data are available from corresponding authors.
